# Control measures following a case of imported Lassa fever from Togo, North Rhine Westphalia, Germany, 2016

**DOI:** 10.2807/1560-7917.ES.2017.22.39.17-00088

**Published:** 2017-09-28

**Authors:** Clara Lehmann, Matthias Kochanek, Diana Abdulla, Stephan Becker, Boris Böll, Anne Bunte, Daniel Cadar, Arno Dormann, Markus Eickmann, Petra Emmerich, Torsten Feldt, Christina Frank, Jochen Fries, Martin Gabriel, Udo Goetsch, René Gottschalk, Stephan Günther, Michael Hallek, Dieter Häussinger, Christian Herzog, Björn Jensen, Felix Kolibay, Michael Krakau, Georg Langebartels, Toni Rieger, Lars Schaade, Jonas Schmidt-Chanasit, Edgar Schömig, Gundolf Schüttfort, Alexander Shimabukuro-Vornhagen, Michael von Bergwelt-Baildon, Ulrike Wieland, Gerhard Wiesmüller, Timo Wolf, Gerd Fätkenheuer

**Affiliations:** 1 German Center for Infection Research (DZIF), Bonn-Cologne, Germany; 2 Department I of Internal Medicine, University of Cologne, Germany; 3 Centre of Integrated Oncology Köln, University of Cologne, Germany; 4 Institute for Virology, Universität Marburg, Germany; 5 German Center for Infection Research (DZIF), Gießen-Marburg-Langen, Germany; 6 Public Health Department Cologne, Germany; 7 Bernhard Nocht Institute for Tropical Medicine, Hamburg, Germany; 8 German Centre for Infection Research (DZIF), Hamburg, Germany; 9 Municipal Hospital of Cologne, Medical Department Holweide, Germany; 10 Department of Tropical Medicine and Infectious Diseases, Center of Internal Medicine II, University of Rostock, Rostock, Germany; 11 Clinic for Gastroenterology, Hepatology and Infectious Diseases, Heinrich Heine University, Düsseldorf, Germany; 12 Department for Infectious Disease Epidemiology, Robert Koch Institute, Berlin, Germany; 13 Department of Pathology, University of Cologne, Germany; 14 Health Protection Authority City of Frankfurt am Main, Germany; 15 University Hospital Frankfurt, Institute of Medical Virology, Germany; 16 Centre for Biological Threats and Special Pathogens, Robert Koch Institute, Berlin, Germany; 17 Department for Clinical Affairs, University of Cologne, Germany; 18 Clinical director University of Cologne, Germany; 19 University Hospital Frankfurt, Department of Infectious Diseases, Germany; 20 Institute of Virology, University of Cologne, Germany

**Keywords:** Lassa fever, hemorrhagic fever, isolation, quarantine, contact precautions, West Africa

## Abstract

In a patient transferred from Togo to Cologne, Germany, Lassa fever was diagnosed 12 days post mortem. Sixty-two contacts in Cologne were categorised according to the level of exposure, and gradual infection control measures were applied. No clinical signs of Lassa virus infection or Lassa specific antibodies were observed in the 62 contacts. Thirty-three individuals had direct contact to blood, other body fluids or tissue of the patients. Notably, with standard precautions, no transmission occurred between the index patient and healthcare workers. However, one secondary infection occurred in an undertaker exposed to the corpse in Rhineland-Palatinate, who was treated on the isolation unit at the University Hospital of Frankfurt. After German authorities raised an alert regarding the imported Lassa fever case, an American healthcare worker who had cared for the index patient in Togo, and who presented with diarrhoea, vomiting and fever, was placed in isolation and medevacked to the United States. The event and the transmission of Lassa virus infection outside of Africa underlines the need for early diagnosis and use of adequate personal protection equipment (PPE), when highly contagious infections cannot be excluded. It also demonstrates that larger outbreaks can be prevented by infection control measures, including standard PPE.

## Introduction

Lassa fever (LF) is an acute viral disease caused by an enveloped RNA virus from the *Arenaviridae* family with a zoonotic reservoir. It is endemic in West Africa, particularly in the four countries of Guinea, Sierra Leone, Liberia and Nigeria [1]. Moreover, cases in Ghana, Mali and Benin [2] have also been reported [3]. Its clinical course ranges from asymptomatic infection to severe haemorrhagic disease [4]. The observed case-fatality rate among hospitalised patients with LF is 15% [5]. Nosocomial infections have also been described [6]. Infections with Lassa virus (LASV) outside Africa are rare. Until March 2016, only 13 imported cases have been reported in the European Union (EU) and the United States (US), seven of which were fatal [7]. All 13 patients had been infected during stays in West Africa. When LF is diagnosed or suspected, adequate infection control measures have to be applied to prevent secondary infections [8].

Here we describe the anti-epidemic measures taken around a case of LF imported from Togo to Cologne, North Rhine-Westphalia, Germany.

## Case report

On 25 February 2016, a previously healthy nurse in his mid-40s who worked in a hospital in Togo, was admitted to the University Hospital of Cologne (UHC), in Germany. Approximately two weeks earlier in Togo the patient had developed fever, malaise, and a sore throat. He had immediately started treatment for suspected malaria, but was admitted to a local hospital 6 days later with ongoing fever and abdominal tenderness. On 22 February, the patient had undergone a diagnostic laparotomy in Togo, which yielded no pathologic result. As the patient’s condition deteriorated continuously, he was medevacked to UHC for further treatment. Upon admission to the intensive care unit, the patient was in septic shock, intubated and mechanically ventilated. The abdominal laparotomy wound discharged clear serous secretions but appeared otherwise inconspicuous. Laboratory tests showed leucocytosis, anaemia, acute renal failure, and liver failure, disseminated intravascular coagulopathy and elevated inflammation parameters (Table 1).

**Table 1:** Results of laboratory tests at different points in time, case of Lassa fever imported from Togo to Germany, 2016

Variable	Reference range (adults)	Upon hospital admission	10 hours after hospital admission
Haematocrit (%)	42.00–50.00	29	17
Haemoglobin (g/dL)	13.5–18.0	9.2	5.5
Reticulocyte count (%)	0.3–1.8	ND	0.9
White cell count (per mm^3^)	4,400–11,300	34,670	31,680
Platelet count (per mm^3^)	150,000–400,000	190,000	115,000
Prothrombin time (%)	70–120	49	< 10
Prothrombin time international normalised ratio	2.0–4.5	1.5	ND
Activated partial-thromboplastin time (sec)	< 36	70	> 120
Fibrinogen (mg/dL)	2.1–4.0	0.8	< 0.5
Sodium (mmol/L)	135–145	141	159
Potassium (mmol/L)	3.6–4.8	6.1	5.5
Chloride (mmol/L)	94–110	111	97
Calcium (mg/dL)	2.04–2.59	1.49	1.15
Phosphorus (mg/dL)	0.81–1.45	2.67	5.84
Magnesium (mg/dL)	0.7–1.1	0.93	1.27
Glucose (mg/dL)	74–109	101	338
Total protein (g/dL)	66–87	43	30
Albumin	35–52	20	19
Alanine aminotransferase (U/L)	< 50	948	918
Aspartate aminotransferase (U/L)	< 50	5,372	4,720
Bilirubine total (mg/dL)	<1.2	1.1	0.8
Creatinine (mg/dL)	0.5−1.1	6.9	5.79
Creatinine kinase (U/L)	< 190	6,383	7,371
Lactic dehydrogenase (U/L)	< 250	11,164	8,239
C-reactive protein (mg/L)	< 5.0	55.7	19.9
Procalcitonin (µg/L)	< 0.1	4.4	1.5
Lactate	< 2.2	21	> 30

ND: Not done.

Results of the blood smears tests were negative for malaria. Therefore no malaria treatment was started. Computer tomography scans of the thorax and abdomen exhibited pleural effusions, slight pericardial effusion and thickening of the bowel wall. There was no indication for a new laparotomy or other reason for a surgery procedure. The patient received broad-spectrum antibiotics and increasing amounts of vasopressor support and epistaxis was treated with a nasal packing. Despite intensive medical treatment the patient’s condition deteriorated continuously and he died ca 10 hours after admission. The clinical diagnosis was septic shock of unidentifiable origin. The pathological post mortem exam revealed a hepatosplenomegaly with signs of septic shock: a congestion of parenchymal organs, acute renal tubular necrosis and bilaterally dilated atria and ventricles.

The post-mortem examination on 29 February did not reveal a clear infectious cause of death; remarkably, no signs of internal bleeding were detected and the corpse was released from hospital and then transported to Rhineland-Palatinate for embalmment for transfer to Togo for intended interment (detailed description in [9]).

**Figure 1:**
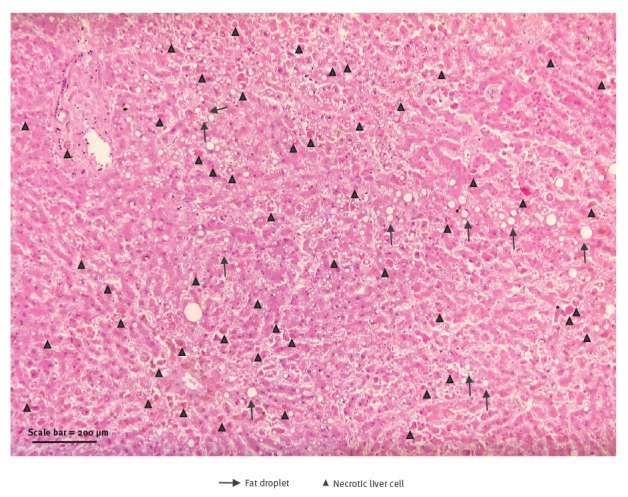
Histopathological image of liver, case of Lassa fever imported from Togo to Germany, 2016

Meanwhile, histological examination of liver sections raised suspicion of acute viral infection likely caused by a haemorrhagic fever virus (Figure 1).

Liver histology showing widespread necrosis without inflammation; individual hepatocytes with acidophilic appearance without any signs of cholestasis and a moderate macrovesicular steatosis with lipofuscin deposition.

H and E stain, magnification 200x.

**Figure 2:**
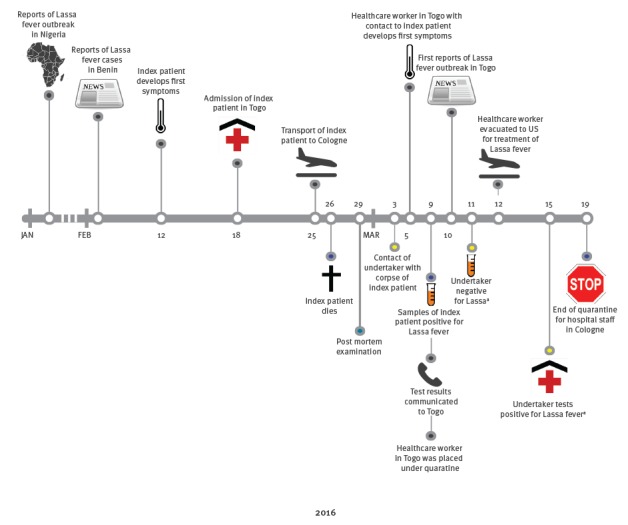
Timeline of events, case of Lassa fever imported from Togo to Germany, 2016

Since viral haemorrhagic fevers (VHF) are endemic in West Africa and the clinical diagnosis was inconclusive, blood and liver samples were sent to the World Health Organization (WHO) Collaborating Centre for Arbovirus and Haemorrhagic Fever Reference and Research (Bernhard Nocht Institute for Tropical Medicine (BNITM), in Hamburg, Germany) for further analyses. The BNITM laboratory reported a positive PCR test for LASV on 9 March 2016 (Figure 2). Other causative agents for viral haemorrhagic diseases such as Crimean-Congo haemorrhagic fever virus, Ebola virus, yellow fever virus, dengue virus and Rift Valley fever virus were negative. The results were communicated immediately to the UHC and to the German health authorities. The corpse was subsequently safely cremated, as required by the German law.

US: United States.

^a^ Details of the secondary transmission are described in [7] and [9].

## Contact tracing and infection control measures

As soon as the diagnosis of imported LF was confirmed on 9 March 2016, a task force was immediately formed. This task force included members of UHC, the local and state public health authorities of North-Rhine Westphalia, and the Permanent Working Group of Competence and Treatment Centers for highly contagious and life-threatening diseases (STAKOB), coordinated by the Robert Koch Institute (RKI), Germany’s federal public health institute.

Based on the literature, the estimated window for potential development of LF in contacts was set at 21 days after last contact [2]. Individuals in North-Rhine Westphalia who had been in contact with the patient, his body fluids or corpse were identified and were defined as primary contact persons. They were interviewed to establish the type of contact and to confirm the presence of symptoms, and instructed about codes of conduct and signs and symptoms to pay attention to. According to national recommendations, four risk categories were defined, with Ia bearing the highest and III bearing the lowest risk of infection (Table 2) [10]. The national recommendations are not specific for LF but are general recommendations for VHF [10]. Risk categories and infection control measures were discussed by STAKOB, UHC and the local public health authority for this LASV case.

**Table 2:** Categorisation of contacts, case of imported Lassa fever from Togo, North Rhine Westphalia, Germany, 2016

Category	Description
**Category Ia**	Cutaneous, percutaneous, needle stick or mucosal exposure to blood, or other body fluids or tissue of the index patient without appropriate personal protection equipment (PPE)
**Category Ib**	Exposure to blood, or other body fluids or tissue of the index patients with appropriate PPE (e.g. nursing and medical staff, laboratory staff, cleaning staff)
**Category II**	Caring, examining diagnostic specimen with appropriate PPE, exposure to clothes, linen or other objects of the index person
**Category III**	Any other kind of contact with the index patient (e.g. staying in the same room)

Adapted from [10].

Infection control measures were applied depending on the respective risk category (Table 3) adapted from [10].

**Table 3:** Types of contacts, risk categories and infection control measures, case of imported Lassa fever from Togo, North Rhine Westphalia, Germany, 2016

Measures	Category			
	Ia Contacts with high risk	Ib Contacts with increased risk	II Contacts with moderate risk	III Contacts with low risk
**Contacts without symptoms**				
Observation, temperature measurement	**+**	**+**	**+**	**-**
General interdiction of work	**+**	**+**	**-**	**-**
Home quarantine	**NA**	**+**	**-**	**-**
Quarantine in hospital	**+**	**-**	**-**	**-**
Blood sampling in case of future assessments	**+**	**+**	**+**	**+**
Virological tests (PCR etc.)	**+**	**+**	**+**	**+**
**Contacts with symptoms (e.g. fever)**				
Observation, temperature measurement	**+**	**+**	**+**	**+**
General interdiction of work	**+**	**+**	**+**	**+**
Home quarantine	**NA**	**NA**	**+**	**+**
High-level isolation in hospital	**+**	**+**	**+ / −**	**+ / −**
Virological diagnostics (PCR etc.)	**+**	**+**	**+**	**+**
Post-exposure prophylaxis	**+**	**+**	**+ / −**	**+ / −**

NA: not applicable; PPE: personal protection equipment.

+ required measure; – not required measure; + / − decision on a case-by-case basis.

Adapted from [10].

Based on the recommendations by Germany’s central institution of biomedicine and public health, the Robert Koch Institute, Berlin, four risk categories were adapted from [10]. Infection control measures were applied depending on the isolation category.

According to national recommendations, contacts classified in category Ib without clinical symptoms were placed in home quarantine. They were instructed to take their temperature twice daily and they were also interviewed for the presence of symptoms twice daily by public health authorities. Based on a discussion by STAKOB, UHC and local public health authorities and following risk-benefit analysis, prophylactic treatment with ribavirin was not provided as the benefit is not clear and adverse effects are frequently observed. Home quarantine was discontinued in the absence of clinical symptoms 21 days after the last contact with the infected patient.

## Laboratory testing

Serum samples from contacts were collected on the day of the first counselling and 4 to 6 weeks later. If any clinical symptoms developed, PCR testing for LASV was performed and category Ib contacts were taken into strict isolation on specialised wards. Lassa testing was undertaken at BNITM, WHO Collaborating Centre for Arbovirus and Haemorrhagic Fever Reference and Research, Hamburg, Germany. LASV RNA was purified from plasma using QIAamp Viral RNA Mini Kit (Qiagen, Hilden, Germany) and detected using an in-house RT-PCR assay targeting LASV S and L RNA segments [11,12]. PCR fragments were sequenced to confirm the diagnosis. Serology for the detection of IgG or IgM antibodies against three different LASV strains (AV, Josiah, Togo 2/2016) was performed with indirect immunofluorescence assays (IIFA) using LASV-infected Vero cells as described previously [12,13]. Sera were tested at a dilution of 1:20.

## Results

Contact tracing identified 62 individuals from different services (medical, laboratory, cleaning, technical, transportation) in North-Rhine Westphalia who had contact with the patient, his body fluids, or corpse in Cologne. Thirty-three contacts were classified in risk category Ib, 17 in category II, and 12 in category III. Notably, all physicians and nurses caring for the patient directly, as well as the pathologists, used appropriate personal protection equipment (PPE). Appropriate PPE included gloves, gowns, facemasks and goggles. However, no specific PPE for haemorrhagic fevers was used. All 33 contacts in category Ib were placed in home quarantine. Primary contacts (n = 55) were offered Lassa serology testing while primary contacts who developed symptoms received LASV PCR testing in addition to serology. Overall, seven contacts developed mostly mild clinical symptoms (low grade fever, respiratory symptoms, or gastrointestinal discomfort) during the incubation period of 21 days. In seven contacts (six of whom were temporarily hospitalised on an isolation ward) LASV infection was ruled out by repeated PCR testing.

However, one secondary infection occurred in an undertaker living in Rhineland-Palatinate, who was classified in category II on 11 March, 2 days after the diagnosis of Lassa fever had become known [9].

Importantly, post-mortem examination of the patient from Togo did not initially reveal an infectious cause of death. PCR results from a sample of the undertaker taken on 11 March were negative for LASV. He became febrile again on 15 March and immunglobuline M (IgM) against LASV and a second PCR were positive on 15 March. He was immediately hospitalised on a high-level isolation unit and had a prolonged course of LF [14].

For LASV serology, serum samples were collected from 55 contacts of the index patient (Table 4). Seven contacts did not provide blood for testing. Except for the undertaker, all other contacts remained negative for IgG against LASV.

**Table 4:** Characteristics and Lassa virus (LASV) laboratory results of primary contact persons of the index patient for whom LASV testing was performed, Germany 2016 (n=55)

Contact category^a^	Ib	II	III	Summary of primary contacts
Number	33	17	5	55
**Sex (n)**				
Female	17	10	3	30
Male	16	7	2	25
Median age, years (IQR)	41 (31–49)	41 (28–45)	29 (28–29)	39 (29–46)
**Type of contact (n)**				
Patient, alive	18	5	0	23
Corpse	9	1	0	10
Body fluids	4	11	1	16
Fixed patient material	2	0	0	2
No direct contact^b^	0	0	4	4
**Profession (n)**				
Physician	9	2	0	11
Pathologist/coroner	3	0	0	3
Nurse	7	1	4	12
Laboratory technician/medical assistant	8	10	0	18
Undertaker	3	0	1	4
Transport personnel	2	3	0	5
Cleaning staff	1	1	0	2
Contacts that developed any symptoms (n)	7	0	0	7
Baseline sera (n)	28	14	5	47
Median days after contact collected (IQR)	14 (14–14)	14 (14–16)	14 (14–14)	14 (14–14)
Follow-up sera (n)	25	11	3	39
Median days after contact collected (IQR)	42 (40–48)	45 (42–48)	40 (40–44)	43 (40–48)
Positive anti-LASV IgM IIFA results/baseline sera tested ^c,d^	0/8	0/7	0/2	0/17
Positive Anti-LASV IgG IIFA results/sera tested^c^ baseline sera; follow-up sera	0/28; 0/25	0/14; 0/11	0/5; 0/3	0/47; 0/39
LASV RNA PCR person’s positive/no. of persons tested ^e^	0/7	0/0	ND	0/7

IIFA: indirect immunofluorescence assay; IQR: interquartile range; LASV: Lassa virus.

^a^ The contact categories are defined in Table 2.

^b^ No direct contact with the index patient (e.g. staying in the same room).

^c^ IgG testing performed in baseline and follow-up serum of contact persons.

^d^ IgM testing was only performed in baseline sera of patients from whom no follow-up serum could be collected.

^e^ Only contact persons that developed any symptoms (respiratory, gastrointestinal, fever) were additionally tested with PCR for the detection of Lassa virus-RNA in serum samples.

## International alert

On the night of 9 March 2016, BNTIM informed RKI, WHO Headquarters, the hospital in Togo where the index case had been working and the US Centers for Disease Control and Prevention (CDC) about the laboratory confirmation of LF in the index case. On 10 March, RKI as national contact point officially reported details of the index case to WHO Regional Office for Europe, the International Health Regulations (IHR) National Focal Points of the US and Togo as well as the European Centre for Disease Prevention and Control (ECDC), the latter via the Early Warning and Response System (EWRS) mechanism. The German secondary case was similarly reported on 16 March, with updates on contact tracing results in the following days.

Following the alert, on 12 March 2016, another secondary case working as surgeon, who had cared for the index patient in Togo and who had become febrile, was evacuated to the US where he tested positive for LF virus on 13 March [15] (Figure 2). He also recovered upon hospitalisation in a specialised treatment centre in the US [15]. WHO produced a comprehensive Environmental Impact Statement on LF in Togo and published on 23 March a disease outbreak news on the German cases [16] while ECDC released a Rapid Risk Assessment on Lassa in West Africa, Germany, and the US on the same day [7].

## Discussion

In a patient transferred from a hospital in Togo, who died a few hours after arrival in UHC, the clinical diagnosis was refractory septic shock without identifiable origin. LASV infection had not been suspected initially, due to unspecific clinical symptoms and because Togo had not been known before as a country endemic for LF. Twelve days after the patient’s death, the diagnosis of LASV infection was established by analysis of post-mortem histological and serological samples. Liver histology showed a widespread necrosis without inflammation. Widespread necrosis of hepatocytes is a hallmark of severe acute viral liver damage, however, the morphological appearance in our case was highly unusual for viral damage by hepatitis B or C virus. The type of necrosis, its widespread distribution and the lack of inflammatory response, were highly suggestive of an acute viral hepatitis of the haemorrhagic fever subgroup.

When these findings became known, contacts with the patient, his body and his specimens, had occurred on numerous occasions in absence of specific precautions required for biosafety level 4 (BSL4) pathogens, as was previously described for other imported cases of LF [17].

Individuals with increased risk exposures included the clinical staff who treated this critically ill patient and the pathologists who performed the autopsy. All of them used standard PPE and no transmission occurred in this group. In this context, it is of note that there was limited contact as the patient died within 10 hours of admission. We excluded asymptomatic or mild LASV infection in 47 contacts, serologically. Asymptomatic or mild infections were identified in up to 80% of contacts of infected individuals in similar situations [4]. Despite the severity of the event, seven contacts did not provide serum for testing and several contacts only provided serum once.

The categorisation of contacts was based on recommendations for VHF in general. However, contact triage should be pathogen-specific as incubation periods, post exposure prophylaxis recommendations therapies, and modes of transmission vary for different pathogens. These are critical considerations for determining quarantine and monitoring [18]. For the case described here, the recommendations were based on a discussion STAKOB, UHC and the local public health authority. While quarantine may prevent further transmission of highly pathogenic viruses, it may also negatively impact the care for persons who develop symptoms not related to LF as the carers can feel fear being at risk of infection. Therefore, healthcare workers have to be prepared to deal with the challenge of recognising and of adequately addressing other conditions than LF in persons under quarantine.

One transmission of LASV was diagnosed in an undertaker in another state who handled the body 6 days after the index person died [9]. Another secondary transmission was diagnosed in a healthcare worker who had previously cared for the index patient in Togo and who was subsequently treated in an American hospital. In 2003, a possible transmission of LASV to a physician in Germany, who was exposed to a patient with LF and who started ribavirin prophylaxis ca 36 hours after exposure, was reported [19]. However, the physician was asymptomatic and nosocomial transmission could not be conclusively demonstrated in this case.

Several important lessons can be learned from the event described here. First, rare but highly contagious infections including VHF should be taken into consideration in patients who present with febrile disease and have recently lived or travelled in sub-Saharan Africa. Malaria is highly prevalent in the areas endemic for LF. The majority of LF cases are reported in the countries of the Mano river basin (Guinea, Sierra Leone and Liberia) and Nigeria [1]. Recent Lassa outbreaks have been reported in Burkina Faso (March 2017), Benin (June 2016), Nigeria (May 2016) and Liberia (May 2016) [20]. Lassa epidemics continually challenge health systems in countries with limited resources and have the potential of creating large epidemics. To develop prevention strategies and improve laboratory diagnostics for LF and other dangerous diseases, an international working group, including health ministries from Guinea, Liberia and Sierra Leone, and other partners, has been set up under the umbrella of WHO [20].

Since co-infections are common, a diagnosis or suspicion of malaria should only rule out the suspicion of LF or another VHF, if patients returning from areas where VHFs could potentially occur, have responded sufficiently to malaria specific treatment. Even if VHFs have not been reported before in the specific area, they should be considered and prompt specific infection prevention measures [21]. With the increase of international travel activities, physicians worldwide will be confronted more frequently with similar situations in the future. Importantly, outbreaks of VHF or other highly infectious diseases in Africa may sometimes only be recognised by diagnosis of cases outside endemic areas.

Second, the use of PPE including gloves, gowns, masks and goggles is effective in prevention of transmission of highly infectious viruses [22]. Whether or not high-level isolation units are required for the treatment of LF is a matter of international debate [23]. Of note, a case of secondary transmission of Ebola virus in Spain in 2014 represented a challenge for both health services and public health authorities. This incident highlighted the need for constant updating and training of professionals in the use of PPE to ensure adequate infection control and protection of individuals [24].

Third, information on highly infectious diseases and education in suitable infection prevention measures should not be limited to healthcare workers, but should also include other professionals such as undertakers or cleaning staff who could potentially be affected.

Fourth, to effectively limit transmission of LF or other highly contagious infections, comprehensive hygiene and emergency plans should be in place. In our case, the existence of a national working group dedicated to the clinical management of these cases (STAKOB) was extremely valuable. There was significant uncertainty on how to deal with specific risk contacts, which needed concerted decisions by the state and local public health authorities.

In conclusion, all exposed healthcare workers (n = 27) in category Ib in Cologne were negative for LASV and one transmission occurred in an undertaker exposed to the corpse in Rhineland-Palatinate. Early diagnosis and management of haemorrhagic fevers and other uncommon communicable diseases require a high level of awareness. Standard infection prevention measures such as wearing gloves, gowns, masks and goggles should be consistently applied when caring for febrile patients with undetermined diagnosis after returning from tropical areas [25].
